# The Tower of Babel of Pharma-Food Study on Extra Virgin Olive Oil Polyphenols

**DOI:** 10.3390/foods11131915

**Published:** 2022-06-28

**Authors:** Maria Lisa Clodoveo, Marilena Muraglia, Pasquale Crupi, Rim Hachicha Hbaieb, Stefania De Santis, Addolorata Desantis, Filomena Corbo

**Affiliations:** 1 Interdisciplinary Department of Medicine, University of Bari “A. Moro”, 70124 Bari, Italy; marialisa.clodoveo@uniba.it (M.L.C.); pasquale.crupi@uniba.it (P.C.); 2 Department of Pharmacy-Pharmaceutical Sciences, University of Bari “A. Moro”, 70125 Bari, Italy; stefania.desantis@uniba.it (S.D.S.); filomena.corbo@uniba.it (F.C.); 3 Biocatalysis and Industrial Enzymes Group, Laboratory of Microbial Ecology and Technology, Carthage University, National Institute of Applied Sciences and Technology (INSAT), BP 676, Tunis 1080, Tunisia; rimhachicha@yahoo.fr; 4 Department of Soil, Plant and Food Sciences (DISPA), University of Bari “A. Moro”, 70126 Bari, Italy; addolorata.desantis@uniba.it

**Keywords:** polyphenols, EVOO, antioxidant and anti-inflammatory properties, health claim, human studies

## Abstract

Much research has been conducted to reveal the functional properties of extra virgin olive oil polyphenols on human health once EVOO is consumed regularly as part of a balanced diet, as in the Mediterranean lifestyle. Despite the huge variety of research conducted, only one effect of EVOO polyphenols has been formally approved by EFSA as a health claim. This is probably because EFSA’s scientific opinion is entrusted to scientific expertise about food and medical sciences, which adopt very different investigative methods and experimental languages, generating a gap in the scientific communication that is essential for the enhancement of the potentially useful effects of EVOO polyphenols on health. Through the model of the Tower of Babel, we propose a challenge for science communication, capable of disrupting the barriers between different scientific areas and building bridges through transparent data analysis from the different investigative methodologies at each stage of health benefits assessment. The goal of this work is the strategic, distinctive, and cost-effective integration of interdisciplinary experiences and technologies into a highly harmonious workflow, organized to build a factual understanding that translates, because of trade, into health benefits for buyers, promoting EVOOs as having certified health benefits, not just as condiments.

## 1. Introduction

### The Tower of Babel Model: A Challenge for Pharma-Food Science Communication on EVOO Polyphenols

Nowadays, there is considerable attention toward the functional properties of extra virgin olive oil (EVOO), the main product obtained from olives, fruits that come from evergreen trees [1,2]. EVOO is a characteristic element of the Mediterranean diet (MD) because of the health-beneficial effects deriving from its chemical composition [3,4,5] as well as its appreciable taste and usefulness in flavoring a large variety of foods.

The overall literature studies report a wide variety of chemical and enzymatic analyses in vitro, in vivo, and ex vivo to reveal the beneficial effects of EVOO polyphenols on human health. EVOO is one of the foods naturally rich in polyphenols and is the main lipid source in MD. Numerous scientific papers and reviews have shown that the potential health benefits of EVOO are correlated to its high content of functional compounds such as polyphenols, tocopherols, carotenoids, sterols, fatty acids, and squalene. This specific chemical composition helps to prevent the incidence of multiple diseases such as cardiac, vascular, neurodegenerative, metabolic, and inflammatory ones [1,6,7,8,9].

In agreement with Visioli et al. [10], there is an urgent need to provide unequivocal scientific evidence that can rationalize EVOO consumption, as an integral and essential part of a balanced diet and healthy lifestyle, to quality of life in terms of prevention and health maintenance. In fact, despite an exorbitant number of papers, only one effect of the EVOO polyphenols has been officially accredited as a health claim by the European Food Safety Authority (EFSA).

The need for new research able to deal systematically with the issue of phenolic compounds present in EVOO and the related actions on human health is confirmed by the fact that many of the requests of authorization to apply health claims on EVOO health effects have received a negative scientific opinion from the EFSA. The latter could be ascribed to different reasons, including the insufficient characterization of food constituent(s), the lack of beneficial physiological effects of the proposed claimed effect, and, most of all, the quality of the studies provided for the scientific substantiation of the claims. Among the numerous olive oil claims that were submitted to EFSA, only one was allowed: “Protection of LDL particles from oxidative damage” [11]. Specifically, the panel based its decision on “a well-conducted and powered study, and two smaller-scale studies that showed dose-dependent and significant effects of olive oil polyphenol consumption (for three weeks) on appropriate markers of LDL peroxidation (oxLDL)”.

Moreover, the EFSA Panel on Nutrition, Novel Foods and Food Allergens (NDA) considered that the quantity of olive oil required to obtain the claimed effect is 20 g (two tablespoons) containing at least 5 mg of hydroxytyrosol and its derivatives (e.g., oleuropein complex and tyrosol). This can reasonably be consumed within a balanced diet, and the proposed wording (although the terminology can be considered excessively technical and not fully understandable for most consumers) reflected the scientific evidence, complying with the criteria for the use of claims specified in the EU Regulation 1924/2006 [11,12].

However, no other health claim on EVOO polyphenols has been approved by the competent authorities to date. The critical and objective analysis of this result reveals that most of the scientific investigations so far aimed at highlighting the health value of polyphenols in EVOO have not met the requirements for the authorization of a novel health claim while denouncing the inappropriate choice of investigative tools and the insufficient evidence provided to establish a cause–effect relationship between the daily consumption of polyphenols in EVOO and the claimed beneficial effect.

This evidence sheds new light on the need to bring together skills belonging to different scientific areas, from food sciences to medical sciences, in a context of skills that still do not communicate effectively due to excessively clear boundaries between the different methods of investigation and specific languages of each discipline. To achieve a better understanding of the real potential beneficial effects of EVOO polyphenols on consumers’ health and well-being, there is a need to create osmotic communication among different scientific areas, building bridges through a clear evaluation of the information coming out from the various methodologies of investigation in each step of health benefits assessment. The failure rate of applications for the approval of health claims concerning EVOO is enormous compared to the intellectual, human, and financial resources spent to prepare the application reports supporting the cause-and-effect relationship. Unfortunately, science, focusing on bridging information deficits about the health value of EVOO polyphenols, has underestimated the need to rework its communication models to overcome regulatory difficulties in favor of valuing the health claims of EVOOs.

Therefore, this circumstance represents clear evidence of the need to point out some aspects concerning the EVOO phenols, such as the mechanisms of action and the laboratory methods used to identify, quantify, and test the antioxidant and anti-inflammatory properties; these analytical tools need to be critically evaluated to verify their method of defining the mechanisms of action and their practical utility in the transfer of information in the subsequent phases of the cause-dose-effect evaluation, when the tests on humans will have to confirm the studies of drug dynamics, pharmacokinetics, and the potential toxicity, to define the effective dose and limitations of use.

However, each research tool has a different efficacy in the health benefit validation process. Most of them need to be harmonized to accelerate the progression of functional food research through a highly active interconnection with related fields.

The challenge for communication across scientific fields, then, is not to discuss which experimental protocol to adopt to improve the health value of EVOO, but to link scientific knowledge and expertise in a transdisciplinary way by making it objective, meaningful, reproducible, and transferable to public health policy authorities.

**Figure 1:**
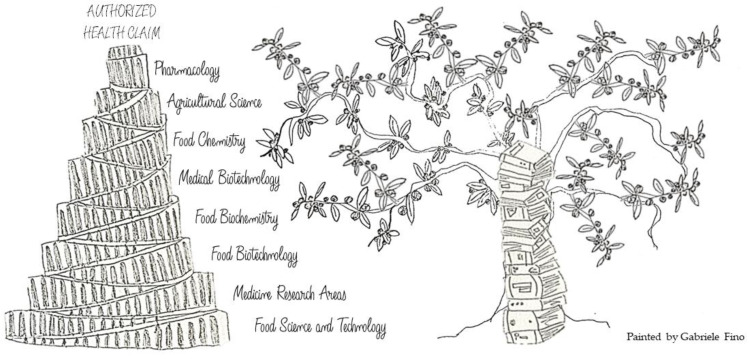
The metaphor of the Tower of Babel of the functional food research sector.

Thus, cross-science intellectual fertilization is needed, overcoming the current stratification of the various scientific fields through a fluid and osmotic communicative process among stakeholders. Herein, we purpose the Tower of Babel as a metaphor for a scientific approach made of different disciplines and backgrounds that need to be integrated by the synthesis of methodological procedures which draw upon more than one scientific area, challenging conventional disciplinary approaches to generate the emerging sector of pharma-food research (Figure 1).

A specialized disciplinary language favors more efficient intra-disciplinary communication but is a tool that excludes outsiders in a particular field. Thus, the Tower of Babel is also a metaphor for different forma mentis. In this regard, a meta-disciplinary awareness is necessary, as the ability to think about the goals, methods, and forms of communication of disciplines harmonizes the focus of disciplinary knowledge and inquiry, and recognizes the roles and constraints imposed by individual disciplines in the goal attainment.

**Figure 2:**
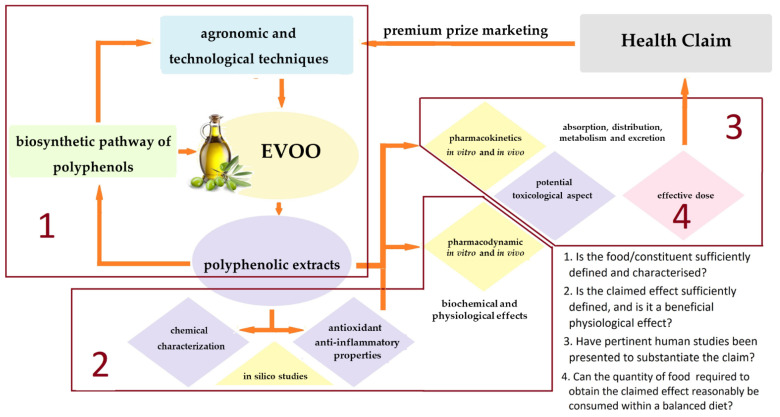
Workflow for pharma-food study on extra virgin olive oil polyphenols.

In this work, we report an overview of the main methodologies adopted by the different disciplinary areas involved in characterizing the polyphenolic and antioxidant profile of EVOOs and evaluating the antioxidant and anti-inflammatory properties potentially useful for obtaining a health claim. This paper aims to suggest a strategic, unique, and efficient integration of cross-disciplinary expertise and technology in a harmonized workflow (Figure 2), organized to build factual understanding that can be translated thanks to industry into consumer health benefits, by marketing EVOOs as having certified health benefits, and not only as seasonings.

## 2. EVOO Polyphenols

### 2.1. Quali- and Quantitative EVOO Polyphenols Characterization: A Dual Communication?

**Figure 3:**
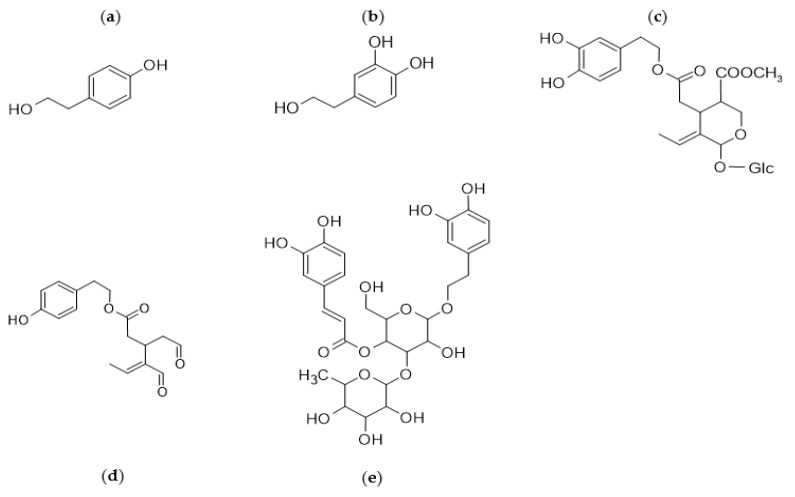
Most abundant polyphenols in olives: (**a**) tyrosol; (**b**) hydroxytyrosol; (**c**) oleuropein; (**d**) oleocanthal; and (**e**) verbascoside.

The most represented chemical classes in *Olea europea* L. trees are mainly classified as nonpolar compounds (present in the lipophilic oil fraction, such as squalene, tocopherols, sterols, and triterpenic compounds) and polar phenolic compounds [1,13]. Among the polyphenolic compounds, the most abundant and studied in olives are tyrosol (TY), hydroxytyrosol (HT), oleuropein (OL), oleocanthal, and verbascoside (Figure 3) [1].

The secondary metabolites from *Olea europea* L. have high biological value, and they are present in different concentrations in the various parts of the olive plant [1]; as such, many of them are present in the derived EVOOs, but they can also be found in the waste products from the production process. In this regard, it should be noted that knowledge of the biosynthetic pathway of EVOO polyphenols is one of the tools that can meaningfully link distant disciplines such as food science, pharmacology, and medicine and accelerate research progress in the field of functional foods.

Understanding the origin of a bioactive compound and how to control food production phases to modulate the concentration of the biomolecule is a crucial step so that scientific evidence of a benefit to human health and the definition of the effective dose can be used as competitive factors in the industrial sector.

Furthermore, knowledge of the biosynthetic pathway of EVOO polyphenols facilitates the standardization of products and the routine use of health claims on the label. These aspects are important for food biochemistry.

All these considerations underline that the qualitative and quantitative characterization of the pool of bioactive molecules that make up food is a priority requirement for the possible authorization of a health claim.

To ensure the transferability of the health claim in the production world, agronomy and food technology work together with olive oil and EVOO producers to choose select extraction methods that reduce the influence of food matrix variability factors while ensuring the presence of bioactive compounds in an effective concentration in a consumable food dose.

Indeed, it should be emphasized that EVOO mechanically extracted from healthy olive fruits is an important source of phenolic compounds whose profile and content depend on several factors, including endogenous [14,15], agronomic [16,17], and technological factors occurring before, during, or after the EVOO extraction process [15,18,19,20,21,22,23,24,25].

The EVOO production chain can lead to the high variability of polyphenol contents in the final products. However, due to rapid advances in scientific research on the EVOO production process, it is now realistic to develop supply chain models to predict the impact of the supply chain on the polyphenol content of consumed products.

The qualitative and quantitative characterization of polyphenols is essential to classify the different commercial categories of olive oil, knowledge of which could also lead to the informed and responsible use of the product for health purposes.

**Figure 4:**
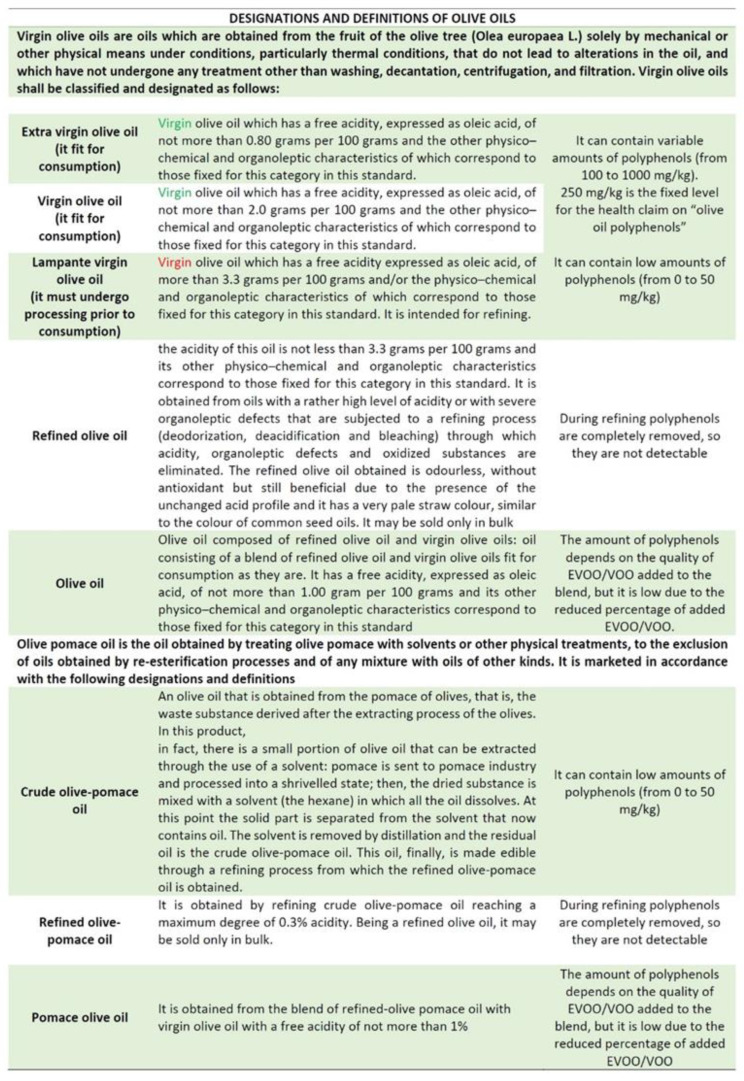
Polyphenol content in the different commercial categories of olive oils and olive-pomace oils [26].

Many countries have subscribed to the standards of the International Olive Oil Council (IOC), which has a United Nations Charter (UN) that sets criteria for different olive oils, their quality, and purity. The indications of the IOC have been implemented by the European Union in the European Regulation 1308/2013, which lists part of the “Designations and definitions of olive oils and olive-pomace oils” in Part VIII (Figure 4) [26].

A search of bibliographic sources through web collections of scientific literature such as PubMed, Scopus, and Google Scholar using “olive oil” and “health benefits” as keywords yielded more than 200,000 published articles, underscoring the considerable attention that academia is devoting to improving the health of olive oil.

Very often the generic term “olive oil” is used, neglecting the correlation between the different commercial categories of olive oils and the content of bioactive compounds whose chemical composition is based on: the mechanical factors of oil extraction; the climatic factors of the harvest; the geographical area; and the methods of conservation/storage of the final product.

Apart from a general reference to the type of oil used, often no chemical characterization has been carried out, not even of the highly variable saponifiable fraction, which is nevertheless within well-defined ranges (monounsaturated and polyunsaturated fatty acids are the subject of specific health claims for EVOOs), nor of the unsaponifiable fraction (in which both polyphenols and tocopherols can be certified with specific health claims).

This gap is likely due to poor communication between the disciplinary sectors involved in olive oil research.

Consequently, the lack of a precise definition of the commercial category used in the experiments and the complete chemical characterization of the matrix and polyphenolic compounds is both an obstacle to the reproducibility of the experiment and to the use of the research results as evidence to support a health claim. Reproducibility is an important principle of the scientific method. It means that a result obtained by an experiment or observational study should be achieved again with a high degree of consistency when the study is repeated using the same methodology by different researchers. Only after one or more successful repetitions should a result be accepted as scientific knowledge. Unfortunately, many experiments today, although published in reputable journals, do not effectively add to the knowledge of the health benefits of EVOO polyphenols. Some of these reports provided confusing or difficult-to-interpret results; however, in the last 10 years, these gaps have been closed [27].

### 2.2. Characterization of EVOO Polyphenols: The Lack of an Official Methodology

Although the EFSA Food Matrix Characterization Panel’s opinion on the approved claim for the ability of polyphenols in olive oil to “protect LDL particles from oxidative damage” is painfully positive, in reality, there is still a gap in terms of the ease of use of the claim on the EVOO label: the lack of an official methodology. After the publication of the approved claim, a great scientific discussion on the subject began. This topic brought together experts in food science and technology, including food chemists and analytical chemists. The HPLC method established by the IOOC for the determination of polyphenols in olive oils has not yet been implemented at the legal level [28].

There is a contrasting need for an analytical method that should be simple, repeatable, and easily adaptable to the conditions of as many laboratories as possible and for the use of high-throughput analytical techniques that are more accurate but also more complicated and that would be mandatory for the identification and quantification of each molecule of the bioactive polar phenols present in each structural form [29,30].

Be that as it may, to date there are no official methods for the determination of total phenols or the most active individual molecules in existing legislation, nor are there any legal limits for their content. As far as the analysis of total phenol content in EVOO is concerned, the most widely used test is the Folin–Ciocalteu colorimetric assay, a well-standardized electron transfer-based method suitable for the routine determination of total phenols in both hydrophilic and lipophilic matrices [31]. It is worth noting that the major limitation of the method is its lack of selectivity, as it leads to the simultaneous determination of all types of phenolic molecules present in the EVOO extract.

In line with previous reports, the EFSA Panel also commented negatively on the usefulness of using the Folin–Ciocalteu assay for the characterization of polyphenols in olive oil. In numerous EFSA opinions, the experts reported that the Folin–Ciocalteu method is not specific for polyphenols because other reducing compounds such as ascorbic acid, sugars, and proteins are also included in the quantification, leading to an overestimation of the actual polyphenol content (see Section 3.1). The total polyphenol content determined with this method is not suitable for the characterization of polyphenols in food.

A lot of elements or compounds (i.e., sugars, proteins, aromatic amines, metals, or other reductones) with high reductive potentials can also interfere with the reagent to give overestimated phenolic concentrations. Finally, even if simple to perform, the Folin–Ciocalteu test is time-consuming (it is necessary to wait for 1.5–2 h before spectrophotometric analysis) [32,33].

In a recent study, however, it was shown how the simple and rapid Folin–Ciocalteu method could give results proportional to the ones obtained by the more laborious and solvent consuming acid hydrolysis-HPLC [33].

In 2009, the International Olive Council (IOC) adopted a high-performance liquid chromatography coupled to the UV/Vis spectrophotometric detector (HPLC-UV) method for the determination of polyphenols in olive oils [34]. Specifically, based on the information about the maximum absorbance values and relative retention times, the method allows up to 27 phenolic molecules to be identified and quantified via converting the sum of the areas of the related chromatographic peaks to tyrosol equivalents (mg of tyrosol/kg of oil). This HPLC-UV determination is certainly characterized by good sensitivity and specificity, but it is also time and solvent consuming [35] and is rather cumbersome due to resolution problems, especially at higher retention times where several isobaric derivatives of secoiridoids appear. Moreover, because of the lack of commercial standards, real external calibration is not performed, which prevents a punctual and reliable identification and quantification [36,37]. To overcome these drawbacks, an analytical method consisting of acidic hydrolysis of bound forms in EVOO methanol/water extract followed by HPLC analysis to indirectly quantify secoiridoids and improve the recognition of lignans, pinoresinol, and acetoxypinoresinol in the total phenolic fraction has been proposed as a reliable alternative thanks to its simplicity and relevance to the bioactive phenolic moieties covered by the claim [38,39]. The method can also allow for assessing the total phenols before hydrolysis according to the IOC official method and can compare their contents before and after the acidic hydrolysis to indirectly investigate the secoiridoidic precursors. However, the issue regarding the correct identification of the phenolic structures persists [12].

To date, reverse-phase (RP) HPLC-MS employing C18 stationary phases has been widely accepted as the main tool in the identification, structural characterization, and quantitative analysis of phenolic compounds in EVOO [32,40]. HPLC-MS/MS methods using triple quadrupole (QqQ) mass analyzers are by far the most proposed ones for the accurate determination of polar phenols in EVOO because of their high sensitivity and selectivity in multiple reaction monitoring (MRM) acquisition modes [6,23]. On the other side, ion-trap mass analyzers (HPLC-MS) can obtain MS spectra which are helpful to establish fragmentation patterns and then elucidate the structure of more complex secoiridoid derivatives.

Even though MS/MS and MS fragmentation are powerful tools for the structural characterization and identification, the low resolution attainable with QqQ and ion-trap instruments sometimes makes the differentiation between isomers (such as open and closed forms of ligstroside and oleuropein aglycones) and isobars (different compounds with the same nominal mass but different elemental compositions, such as oleacinic and oleocanthalic acid) difficult. For these reasons, high-resolution mass spectrometry (HRMS, such as TOF, Q-TOF, Orbitrap, and FTICR), which provides excellent mass accuracy and measurements of the correct isotopic pattern, especially when combined with tandem mass spectrometry experiments, appears as the best choice to achieve definitive characterization and identification of EVOO polyphenols [41,42,43]. In many cases where mass spectral data are insufficient to establish a definitive structure for these complex phenolic compounds, nuclear magnetic resonance spectroscopy (NMR) is a powerful complementary technique for the structural assignment [44,45]. Further attempts have been used in recent years with a special coupling technique, LC-NMR [46,47,48].

In recent years, ultra-high-performance LC (UHPLC), either using sub-2 μm particle packed columns or porous-shell columns, has opened new possibilities for improving the analytical methods for complex sample matrices such as EVOO, being able to achieve 5- to 10-fold faster separations than with conventional HPLC, while maintaining or even increasing resolution [49,50,51]. UHPLC methods can be considered more cost-effective because they typically consume around 80% less organic solvents than conventional HPLC methods. Today, UHPLC coupled to MS and MS/MS is one of the most widely employed techniques in EVOO phenols analysis because it is typically less affected by possible matrix effects [30,52].

**Figure 5:**
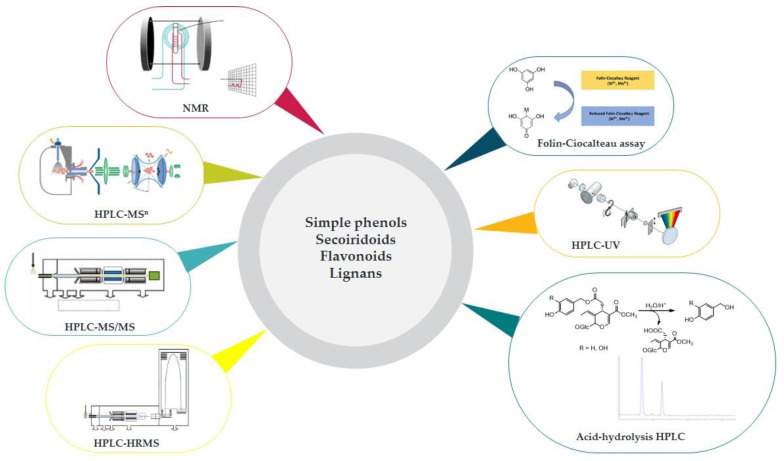
Methods of analysis for polyphenols in EVOO extracts.

Figure 5 graphically summarizes the analytical and colorimetric procedures adopted for the qualitative and quantitative evaluation of EVOO polyphenols.

## 3. EVOO Polyphenols Antioxidant and Anti-Inflammatory Properties

### 3.1. Chemical Antioxidant EVOO Polyphenols Assays: A Preliminary Investigation

Antioxidant assays can be classified concerning different approaches, such as:

- The type of antioxidant measured (e.g., lipophilic, hydrophilic, enzymatic, and non-enzymatic);
- The character of assay medium (e.g., aqueous, and organic solvent, direct or indirect, in situ and ex situ);
- The type of assay reagent (e.g., radical and non-radical initiated reactions);
- The mechanism of action.

Depending upon the mechanism of reactions involved, the antioxidant capacity assays can roughly be classified into two types of reaction-based assays:

1. Single electron transfer (SET);
2. Hydrogen atom transfer (HAT).

The SET mechanism involves a redox reaction with an oxidant which changes color when reduced, as an indicator of reaction endpoint, i.e., when electron transfer has stopped. The degree of color change is correlated with the sample antioxidant concentrations.

SET-based assays include the total phenols assay by:

- Folin–Ciocalteu reagent (FCR);
- Trolox equivalence antioxidant capacity (TEAC);
- 2,2-Azino-bis(3-ethylbenzothiazoline-6-sulfonic acid) assay (ABTS^+^);
- Ferric ion reducing antioxidant power (FRAP);
- CUPRAC: “total antioxidant potential” assay using a Cu(II) complex as an oxidant;
- 2,2-Diphenyl-1-picrylhydrazyl radical scavenging capacity assay (DPPH˙);
- Fast Blue BB diazonium salt (FBBB).

These assays provide a relative measure of antioxidant activity, but often the radicals scavenged have little relevance to those present in biological systems [53]. However, these total antioxidant activity assays in test tubes do not necessarily reflect the cellular physiological conditions and do not consider the bioavailability and metabolism issues. In addition, the mechanisms of action of antioxidants go beyond the antioxidant activity of scavenging free radicals in disease prevention and health promotion [54].

Instead, HAT assays involve a synthetic radical generator, an oxidizable probe, and an antioxidant; they apply a competitive reaction scheme, in which the antioxidant and substrate compete for thermally generated peroxyl radicals through the decomposition of azo compounds. These assays include:

- Inhibition of induced low-density lipoprotein autoxidation;
- Oxygen radical absorbance capacity (ORAC);
- Total radical trapping antioxidant parameter (TRAP);
- Crocin bleaching assays [55].

It is worth noting that both SET and HAT reaction-based assays measure the radical scavenging capacity instead of the preventive capacity of EVOO tout court [56,57]. In particular, SET-based assays measure the antioxidant reducing capacity, while HAT-based assays quantify hydrogen atom donating capacity [58]. Usually, a strong relationship between phenolic compounds in the EVOO extracts and the antioxidant capacities is observed, allowing it to concluded that they can act as effective radical chain-breaking antioxidants and that all the methods tested are suitable for determining the antioxidant capacity of phenolic compounds in olive oil [51,57,59].

However, it is necessary to emphasize that the assays described herein are affected by a series of drawbacks, which have opened a serious debate about their reliability and the difficulty of comparing their heterogeneous results to perform quality control for antioxidant products in the food and nutraceutical industry [57,59]; to give an idea, the best method for determining the antioxidant capacity of olive oil is sometimes referred to as ABTS and sometimes as ORAC. At first, these methods are strictly dependent on the experimental conditions, for instance, the pH, which plays a fundamental role in the reducing capacity of antioxidants. Indeed, the antioxidant assays are carried out at acidic (FRAP), neutral (TEAC), or basic (FCR) conditions; at acidic conditions, the reducing capacity may be suppressed due to protonation on antioxidant compounds, whereas at basic conditions, proton dissociation of phenolic compounds would enhance a sample reducing capacity. Another issue regards the substrate involved: HAT-based assays have a mechanistic similarity to lipid peroxidation, but under the assay conditions, the concentration of the substrate (in this case the probe) is often smaller than the concentration of antioxidants (i.e., polar phenols). This is in contradiction with the real situations: in food systems such as EVOO, the antioxidant concentration is much smaller than that of the substrate (e.g., lipid). Then, the antioxidant assays are usually carried out in a controlled manner in a homogeneous solution with an artificial oxidant or radical precursor added to initiate the reaction, whereas in a real food lipid system the reaction occurs without an added radical initiator or oxidant. Instead, the reaction is initiated by light, metal ions, or heat during food processing or storage. Moreover, it is often a heterogeneous mixture (as in food emulsions), and the phase distribution of antioxidants will be critical for its effectiveness [55,60]. Therefore, to have a more realistic assessment, the measurement of the antioxidant capacity of polyphenols that are mixtures, multifunctional, or are acting in complex multiphase systems such as EVOO must be conducted using a combination of a few assays (minimum three) involving different chemical reactions. Of course, this is time-consuming and expensive, since many reagents are needed [61]. A further controversial concern is that antioxidant capacity assays are strictly based on chemical reactions in vitro. They bear no similarity to biological systems and any claims about the bioactivity of EVOO based solely on ORAC, TEAC, FRAP, etc., would be exaggerated, unscientific, and out of context because they do not report any data about whether the measured antioxidants have any biological role [62,63]. Moreover, these assays do not take into account the bioavailability, in vivo stability, retention of antioxidants by tissues, cellular uptake, transport process, and reactivity in situ. Thereby, none of these assays have much value in terms of whether a source of antioxidants provides antioxidant protection in biological systems except in situations where biological fluids, collected before and after EVOO consumption, for example, are applied in the chemical assays, because in this way the gathered data would tell a story about whether antioxidants are assimilated into the bloodstream in a form that can reduce free radical damage. On the other side, the current antioxidant assays have been reported to possess several strengths, such as a simple procedure, rapid analysis time [55,64], cheap reagents, good correlation with bioactive compounds (phenols and flavonoids), reproducibility [65], and the use of simple instrumentation. These strengths are generally good, but are not strong enough to overcome the aforementioned limitations and to support the efficacy and reliability of these assays as they are seriously flawed.

Recent methodologies adopted to study the antioxidant and anti-inflammatory profile, such as membrane-mimicking systems (liposomes, micelles, etc.) and the activation of cellular antioxidant responses, via Nrf2-Keap1 cascades, could provide a valuable model to study the properties of EVOO polyphenols. In particular, the erythroid nuclear factor 2-like transcription (Nrf2) pathway, which can direct the expression of antioxidant and cytoprotective genes, is the focus of worldwide research. Its activity is induced by exposure to oxidative or electrophilic stresses, including so-called indirect antioxidant compounds. Therefore, special clinical attention is being paid to natural compounds that modify Nrf2 activity, whose pharmacological applications in health and disease are being investigated [66,67].

Generally, the widespread use of antioxidant assays as rapid screening tools rather than as chemical reactions to measure kinetics and determine mechanisms are largely responsible for the inconsistencies, inaccuracies, and controversies in the scientific antioxidant literature, in medicine, and in the popular press.

Regardless, even though alternative and more specific methods have recently been proposed [68,69,70,71], to date a universal and validated protocol for the determination of antioxidant capacities of polar phenols in EVOO as well as other biological samples is still lacking [55].

### 3.2. In Vitro Antioxidant and Anti-Inflammatory EVOO Polyphenols Screening

Inflammation constitutes one of the biological responses of body tissues to nocive stimuli, such as pathogens, injuries, and irritants. It is habitually accompanied by pain, redness, heat, and swelling. Two types of inflammation have been distinguished: acute and chronic inflammation [72]. To begin with, acute inflammation is short-term and only takes a few days to disappear. It results from a cut or injury in the skin, bronchitis, or a sore throat. On the contrary, chronic inflammation is long-term, during which the inflammatory response can firstly damage cells, tissues, and organs and thereafter damage DNA and kill tissues. Consequently, it is generally responsible for the development of several diseases such as cancer, cardiovascular and neurodegenerative diseases, type 2 diabetes, obesity, and asthma [73,74,75]. Non-steroidal anti-inflammatory drugs (NSAIDs) constitute a potent anti-inflammatory drug for the management of inflammatory conditions. However, they are toxic and have secondary effects on human health [76]. For these reasons, the search for natural products with anti-inflammatory activity has been the subject of several studies. Among them, EVOO phenolic compounds have been well proven for their anti-inflammatory activity [7,77,78,79,80,81]. In this regard, in our previous work, we extensively reported and discussed the close correlation between the chemical characterization of EVOO polyphenols, anti-inflammatory potential, and biological activities in human studies [80].

The anti-inflammatory activity has been evaluated using different methods (see Section 3.2.1 and Section 3.2.2).

#### 3.2.1. Inhibition of Protein Denaturation Assay

Considering that the denaturation of protein is linked to the occurrence of inflammatory diseases such as diabetes and cancer, measuring the percentage inhibition of protein denaturation by a substance is useful in predicting the anti-inflammatory effect. The substance to be tested prevents the process of denaturing proteins in relation to the dimension of its anti-inflammatory activity. For that, in this method, either egg albumin or bovine serum albumin (BSA) can be used as the protein.

A volume of egg albumin or BSA was mixed with a sample extract to be tested in phosphate-buffered saline. After 15 min of incubation in a 37 °C water bath, the reaction mixture was heated at 70 °C for 5 min to denaturate the proteins. Then, the turbidity was measured spectrophotometrically at 660 nm. Distilled water or phosphate buffer solution was used as the control in place of extracts to be tested. The percentage inhibition of protein denaturation was calculated using the following formula:

% Inhibition of denaturation = (1 − A1/A2) × 100.

where A1 is the absorbance of the test sample and A2 is the absorbance of the control sample.

#### 3.2.2. Membrane Stabilization Method

The lysis of the lysosomal membrane with releasing of its constituents is considered one of the inflammation results. Consequently, the anti-inflammatory effect of a sample was estimated by its ability to stabilize the lysosomal membranes and thus prevent the release of lysosomal components such as enzymes.

For testing the anti-inflammatory activity, studies have used human red blood cells (HRBCs) as their membranes resemble those of lysosomes. HRBCs could be hemolyzed either by their treatment in a hypotonic solution or by heat [82].

a. Membrane hemolysis induced by hypotonic solution

Hyposaline solution [83] and distilled water [82] were used as hypotonic solutions to favor HRBC hemolysis. In this method, erythrocyte suspension was firstly prepared from human blood and then homogenized in hypotonic solutions with the sample to test. The mixture was then incubated at 37 °C for 30 min and, after its centrifugation at 3000 rpm for 20 min, the hemoglobin content in the supernatant liquid was determined spectrophotometrically at 560 nm.

For a control test, phosphate buffer solution was used in the place of the sample to test.

The percentage of red blood cell membrane stabilization or protection was calculated by the following equation:

Percentage protection= 100 − (A2/A1) × 100

where A1 = absorption of the control and A2 = absorption of the test sample mixture.

b. Heat-induced hemolysis

In this case, the membranes of HRBCs were lysed due to heat treatment. Briefly, the blood cell suspension and the test sample were homogenized in an isotonic solution, such as phosphate buffer, then maintained in a water bath under shaking for 20 min at 54 °C or 30 min at 60 °C according to Gunathilake’s experimental procedure [76]. After centrifugation (2500 rpm for 3 min or 3000 rpm for 5 min), the absorbance of the supernatant was measured spectrophotometrically at 540 nm or 560 nm.

In the control test, a phosphate buffer solution was used. The percentage inhibition of hemolysis was calculated as follows:

Percentage inhibition = (1 − A2/A1) × 100

where A1 = absorption of the control and A2 = absorption of the test sample mixture.

#### 3.2.3. Assay of Cyclooxygenase and 5-Lipooxygenase Inhibition

During an inflammation, arachidonic acid could be metabolized either by the cyclooxygenase (COX) pathway producing prostaglandins and thromboxane A2 or by generating eicosanoids and leukotrienes through the 5-lipoxygenase (5-LOX) pathway [84]. For this reason, anti-inflammatory activity is attained by suppressing the production of prostaglandins and leukotrienes via COX and 5-LOX pathways inactivation.

By focusing on lipoxygenase inhibition assay, this method consists of determining the percentage of LOX activity inhibition in the presence of an anti-inflammatory substance. For that, linoleic acid and soybean lipoxygenase were used as the substrate and enzyme, respectively [76]. Other studies have used lipoxidase and human recombinant lipoxygenase as an enzyme [85].

Briefly, an aliquot of the test sample (10 μL) was homogenized with 20 μL of soybean lipoxygenase solution (167 U/mL) in a sodium phosphate buffer (100 mM; pH 8.0) and then incubated at 25 °C for 10 min. Thereafter, the absorbance was measured spectrophotometrically at 234 nm after the addition of 10 μL of the linoleic acid substrate. In the control sample, the test sample was replaced by a phosphate buffer solution [84].

The percentage of LOX activity inhibition was calculated as follows:

Percentage inhibition = (1 − A2/A1) × 100

where A1 = absorption of the control and A2 = absorption of the test sample mixture.

## 4. In Vitro EVOO Polyphenols Biological Screening: A Point about the Potential Mechanism of Action

The connection between oxidative stress and inflammation has been laid out by many authors. The pathogenic role of mixed advanced glycoxidation products (AGE) and advanced lipid peroxidation products (ALE) generated during oxidative stress and their adducts with cell biomolecules, such as proteins and nucleic acids, in several chronic inflammatory and autoimmune diseases are well documented [86].

The scientific literature reports a wide range of beneficial effects of EVOO polyphenols which are united by an etiology based on oxidative stress and chronic inflammation. Oxidative stress is viewed as an imbalance between the production of reactive oxygen species (ROS) and their elimination by protective mechanisms, which can lead to chronic inflammation. EVOO polyphenols, based on previous evidence, can be useful as an adjuvant therapy for their potential anti-inflammatory effect, associated with antioxidant activity, and the inhibition of enzymes involved in pathways of inflammatory disorders. EVOO polyphenols provide several high biological properties, such as antioxidant [87,88,89], anti-inflammatory [90,91,92], and anti-microbial activities [93,94], which are partially associated with the ability of these natural compounds to scavenge free radicals.

The beneficial effects of EVOO phenolic compounds for human health have been recognized by the American Food and Drug Administration (FDA) [95] and by EFSA, which, as previously reported, advises consumption of about 20 g of EVOO daily. Additionally, in November 2018, the FDA declared the possibility of introducing the “qualified health claim” on EVOO bottle labels.

In this section, we will focus on the antioxidant and anti-inflammatory effects of EVOO polyphenols as they have been the subject of considerable research interest in recent years.

Oxidative stress is the main cause of human diseases [88]. It results from the excessive production of reactive oxygen species (ROS) and/or the low physiological activity of antioxidant defenses against these free radicals. The high production of ROS is responsible for the damage to cell biomolecules such as lipids, DNA, and proteins, thus promoting the appearance of various diseases including cancer, respiratory, cardiovascular, neurodegenerative, and digestive diseases.

To exhibit its antioxidant activity and protect the human body against the previously mentioned diseases, phenolic compounds can act as radical scavengers, chain breakers, or metal chelators. The low redox potentials of polyphenols allow them to reduce highly oxidizing free radicals by chelating metal ions [96]. Moreover, polyphenols can inhibit the activity of enzymes implicated in ROS production and improve the antioxidant defenses system of the cell by acting on gene expression and transduction and enzyme activities [97].

In addition to oxidative stress, the disruption of the balance between pro-inflammatory and anti-inflammatory molecules occurring during an inflammation causes many pathologies [98]. The antioxidant and anti-inflammatory activities of EVOO polyphenols are essentially related to their ability to attenuate reactive oxygen species (ROS), destroy carcinogenic metabolites, and act negatively on the inflammatory processes. Several studies have revealed the antioxidant and anti-inflammatory activities of EVOO polyphenols and their protective effects against several diseases [81,89,99,100,101,102,103,104]. The antioxidant activity of EVOO is not necessarily related to its high content of total phenols. Each phenolic compound has its antioxidant power which is related essentially to its chemical structure [104].

## 5. In Silico Studies on EVOO Polyphenols

Molecular docking methods can be considered the first step to investigating the molecular binding properties of EVOO phenolic compounds to bind to, for example, epigenetic enzymes. Molecular docking results suggest that flavonoids, secoiridoids, and glucosides may bind particularly strongly to epigenetic regulators.

The in silico analysis, thanks to public and private molecular databases which contain the primary structure of any gene or protein of biological interest in humans and in various animal species, including three-dimensional structures on which to carry out computer experiments, allows cellular or physiological processes, even complex ones, to be simulated in a static or dynamic way, allowing the kinetics and tissue distribution of a bioactive molecule to be predicted based on a few experimental points. With this type of technique (molecular docking), it is possible, for example, to verify whether the bioactive molecules isolated from food can interact with the biological target. Computer modeling is widely used to compare the chemical characteristics of bioactive compounds on specific molecular targets, identified thanks to screening strategies involving the use of in vitro or in vivo experimental models. These methodologies allow considerable savings in terms of time and work, favoring a more targeted and rational design of biologically active molecules. For these reasons, the nutraceutical industry has been equipped, for several years now, with groups of bioinformaticians and chemists in charge of identifying the biological targets of bioactive molecules.

ROS and reactive nitrogen species (RNS) are the major reactive species causing oxidative damage in the human body. To counteract their assault, living cells have a biological defense system composed of enzymatic antioxidants that convert them to harmless species. In contrast, no enzymatic action is known to scavenge them. Therefore, the burden of defense relies on a variety of nonenzymatic antioxidants such as vitamins and other phytochemicals that, due to their favorable oxidation potential, have the property of scavenging oxidants and free radicals [60]. In this context, polyphenols are compounds of great interest, having antioxidant properties which derive from several potentially synergistic mechanisms such as radical scavenging, hydrogen atom transfer, singlet oxygen quenchers, and metal-chelating activity [105,106,107]. As radical scavengers or chain breakers, they act by donating hydrogen radicals to alkoxyl and peroxyl radicals formed during the initiation step of lipid oxidation, slowing down the total rate of autoxidation. The presence of catechol moiety (o-diphenols as oleuropein derivatives) stabilizes the phenoxyl radical through an intramolecular hydrogen bond; moreover, they can bind metals, preventing the lipid autoxidation related to EVOO shelf life [40,107].

## 6. Conclusions

Although the scientific literature reports many studies related to the chemical and biological characterization of polyphenols in EVOOs to reveal the functional properties of polyphenols in extra virgin olive oil on human health, only one effect of EVOO polyphenols has been formally approved by the EFSA as a health claim. The validity of the experimental protocols adopted is indisputable, but the scientific communication of the results is strictly limited to the specific knowledge and skills of the research groups involved in the studies. This generates a stratified and non-osmotic scientific communication among researchers to the detriment of the exploitation of the beneficial effects of EVOO polyphenols on human health. In this context, we suggest the model of the Tower of Babel as an opportunity to challenge scientific communication that can favor scientific contamination between the different fields of investigation involved, building bridges through the transdisciplinary analysis of the data of the different investigative methodologies in each stage of the assessment of health benefits. The main objective of this work is to propose an integrated, strategic, and easily transferable scientific communication between the parties that promote the health value of EVOO polyphenols by applying them to health claims.

Since we are certain that fluid communication between scientific knowledge can generate important results in enhancing the health power of EVOO polyphenols, we hope that the Tower of Babel model can be a starting point for the enhancement of scientific results from research related to the food sector and raise awareness among the authorities involved in favor of the use of healthy foods.
